# A Rare Case of Synchronous Glioblastoma and Central Neurocytoma: An Unreported Occurrence

**DOI:** 10.7759/cureus.116460

**Published:** 2026-09-17

**Authors:** Adon Khachik, Wenze Niu, William Nguyen, Shiv Bhanu, Amanda Allen

**Affiliations:** 1 Department of Radiology, Riverside Community Hospital, HCA Healthcare, Riverside, USA; 2 Department of Internal Medicine, Harbor–University of California, Los Angeles Medical Center, Los Angeles, USA

**Keywords:** central neurocytoma, glioblastoma, neuroimaging, primary brain tumors, synchronous tumors

## Abstract

Glioblastoma and central neurocytoma are primary central nervous system tumors with distinct radiologic and histopathologic characteristics. This report presents the case of a 39-year-old male with synchronous glioblastoma and central neurocytoma, an exceptionally rare diagnosis. The patient presented with a seven-day history of headache, nausea, and vomiting, prompting neuroimaging that revealed two supratentorial lesions with imaging and pathologic findings that favored independent tumorigenesis. This case highlights the distinctive imaging features of synchronous glioblastoma and central neurocytoma and emphasizes the role of multimodal imaging and pathologic correlation in establishing the diagnosis.

## Introduction

Glioblastoma is a rapidly progressive, poorly differentiated malignant astrocytic tumor characterized histologically by necrosis and microvascular proliferation. Two clinicopathologic entities have historically been described, namely, primary (de novo) glioblastoma, which accounts for approximately 95% of cases, and secondary glioblastoma arising from malignant transformation of a lower-grade astrocytoma [1]. Although these entities share similar histopathologic features, they are genetically distinct. According to the 2021 World Health Organization (WHO) Classification of Tumors of the Central Nervous System, glioblastoma is defined as an isocitrate dehydrogenase (IDH)-wildtype diffuse astrocytic tumor and may be diagnosed based on molecular alterations, including *TERT* promoter mutation, epidermal growth factor receptor (EGFR) amplification, and combined gain of chromosome 7 with loss of chromosome 10, even in the absence of classic histologic features [2]. This molecularly driven classification enhances diagnostic precision and facilitates individualized treatment strategies based on tumor-specific genetic profiles. Glioblastoma is the most common primary malignant brain tumor in adults, accounting for approximately 49% of cases, while representing approximately 5% of pediatric brain tumors [3]. Incidence increases after the age of 40 years and peaks between 75 and 84 years of age [3]. Treatment generally consists of surgical resection followed by radiotherapy or chemotherapy [4]. Despite maximal efforts, the prognosis remains poor, with median survival remaining approximately 12-15 months, with a five-year survival rate below 10% [4].

Imaging findings of glioblastoma are best characterized by an irregular enhancing rim of tumor surrounding a heterogeneous central necrotic core. It is typically accompanied by a peritumoral T2/fluid-attenuated inversion recovery (FLAIR) hyperintense signal representing adjacent vasogenic edema and/or tumoral infiltration. Additional imaging findings include cystic degeneration, hemorrhage, fluid-debris levels, and flow voids [5]. Glioblastoma most commonly arises within the supratentorial white matter, particularly in the frontal lobe. On CT, it typically appears as a poorly marginated isoattenuating or hypoattenuating mass with hemorrhagic foci and a hypodense center suggestive of necrosis [5].

Central neurocytoma is a low-grade neuronal tumor classified as central nervous system (CNS) WHO grade 2 under the 2021 WHO criteria [2]. It primarily affects younger adults between 20 and 34 years of age and accounts for approximately 0.5% of all brain tumors and 10% of intraventricular tumors [6]. It classically arises within the lateral ventricle and is attached to the septum pellucidum. Imaging features typically include a mixed solid-cystic intraventricular mass with mild-to-moderate heterogeneous enhancement [7]. Central neurocytoma frequently presents with headache, seizures, or, less commonly, intraventricular hemorrhage. Due to its intraventricular location and often large size at presentation, acute obstructive hydrocephalus may be the initial manifestation. Central neurocytomas are believed to arise from neuronal cells, neural progenitor cells, neural stem cells, or other multipotent precursor cells [8]. Treatment options typically include surgical resection, with radiation therapy reserved for recurrent or high-grade tumors [9]. Ten-year survival rates up to 83% have been reported [9].

This case discusses the highly unusual synchronous presentation of a glioblastoma and central neurocytoma occurring in a 39-year-old male. Unlike a previously reported case suggesting a shared clonal origin, the findings in our patient favor independent tumorigenesis [10].

## Case presentation

A 39-year-old male with no significant past medical history presented to the emergency department with seven days of worsening nausea, vomiting, and right-sided headache. Physical and neurological examinations were unremarkable. Initial noncontrast CT of the head demonstrated a large intra-axial hypoattenuating mass in the right posteromedial temporal lobe measuring 5.4 × 3.4 × 4.3 cm (Figure 1). An additional heterogeneous, partially calcified intraventricular mass was present in the body of the left lateral ventricle, measuring up to 4.0 cm and abutting the septum pellucidum (Figure 1).

**Figure 1:**
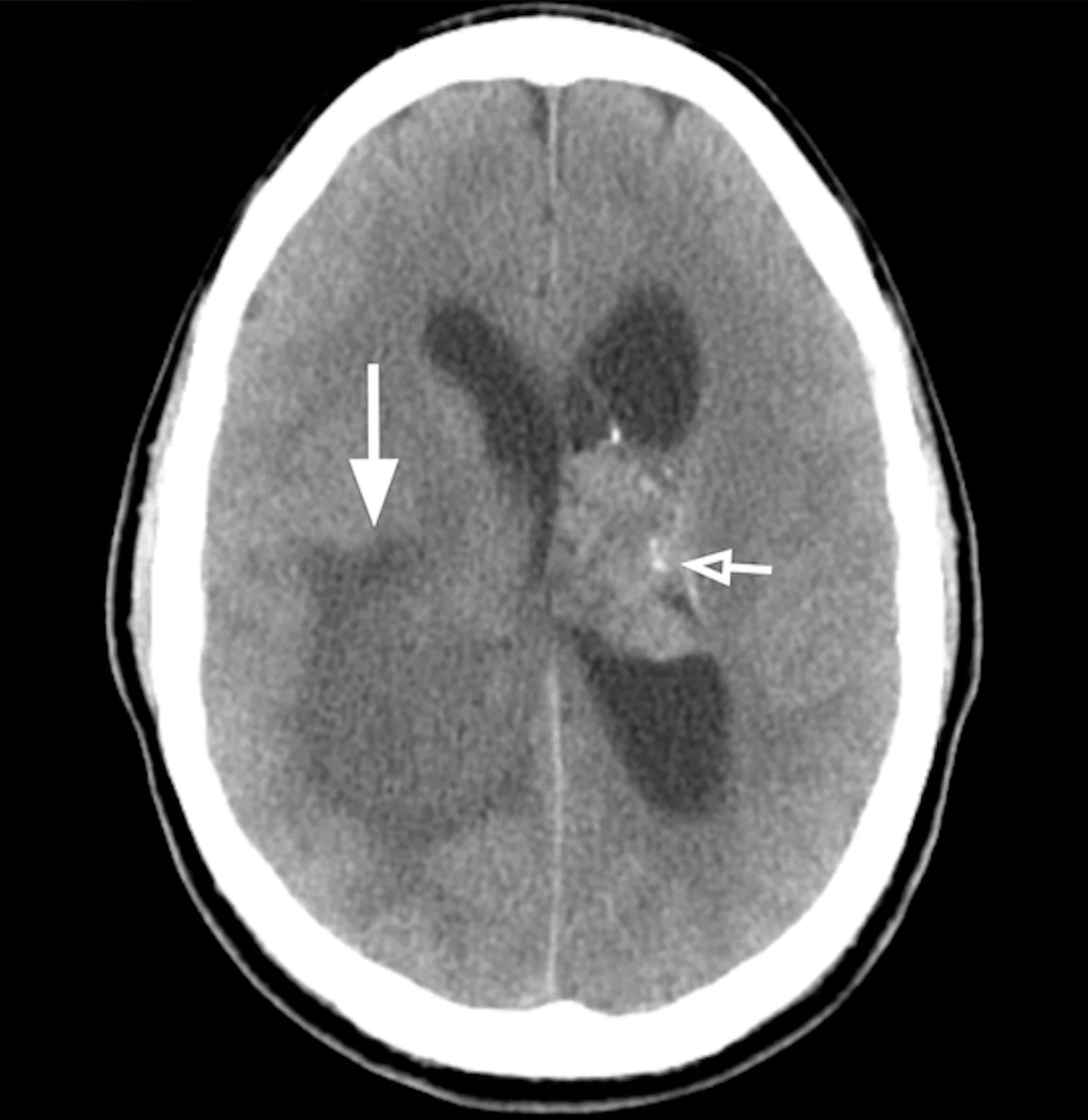
Axial non-contrast CT of the brain. There is a nonspecific ovoid hypoattenuating lesion in the right temporal lobe with surrounding perilesional hypodensity (closed arrow). Although these imaging findings are nonspecific, subsequent imaging and histopathologic findings were consistent with a high-grade glioblastoma. An additional heterogeneous lesion centered within the lateral ventricle demonstrates scattered foci of calcification (open short arrow) and was ultimately diagnosed as a central neurocytoma.

An MRI of the brain with and without contrast was subsequently performed, demonstrating a solid, heterogeneous, enhancing intra-axial mass in the right medial temporal lobe with central nonenhancing mixed-signal-intensity components and small areas of diffusion restriction (Figures 2, 3). The adjacent perilesional FLAIR hyperintensity likely represented peritumoral infiltration and vasogenic edema (Figure 4). Additionally, there was regional mass effect on the right dorsal midbrain and right posteromedial thalamus, with findings concerning for early ventricular entrapment (Figure 2). The intraventricular mass demonstrated a microcystic morphology with subtle heterogeneous enhancement and subtle diffusion restriction (Figures 2-4). Multiple specialties were consulted, including neurosurgery, neurology, and hematology, and a decision was made to proceed with a biopsy.

**Figure 2:**
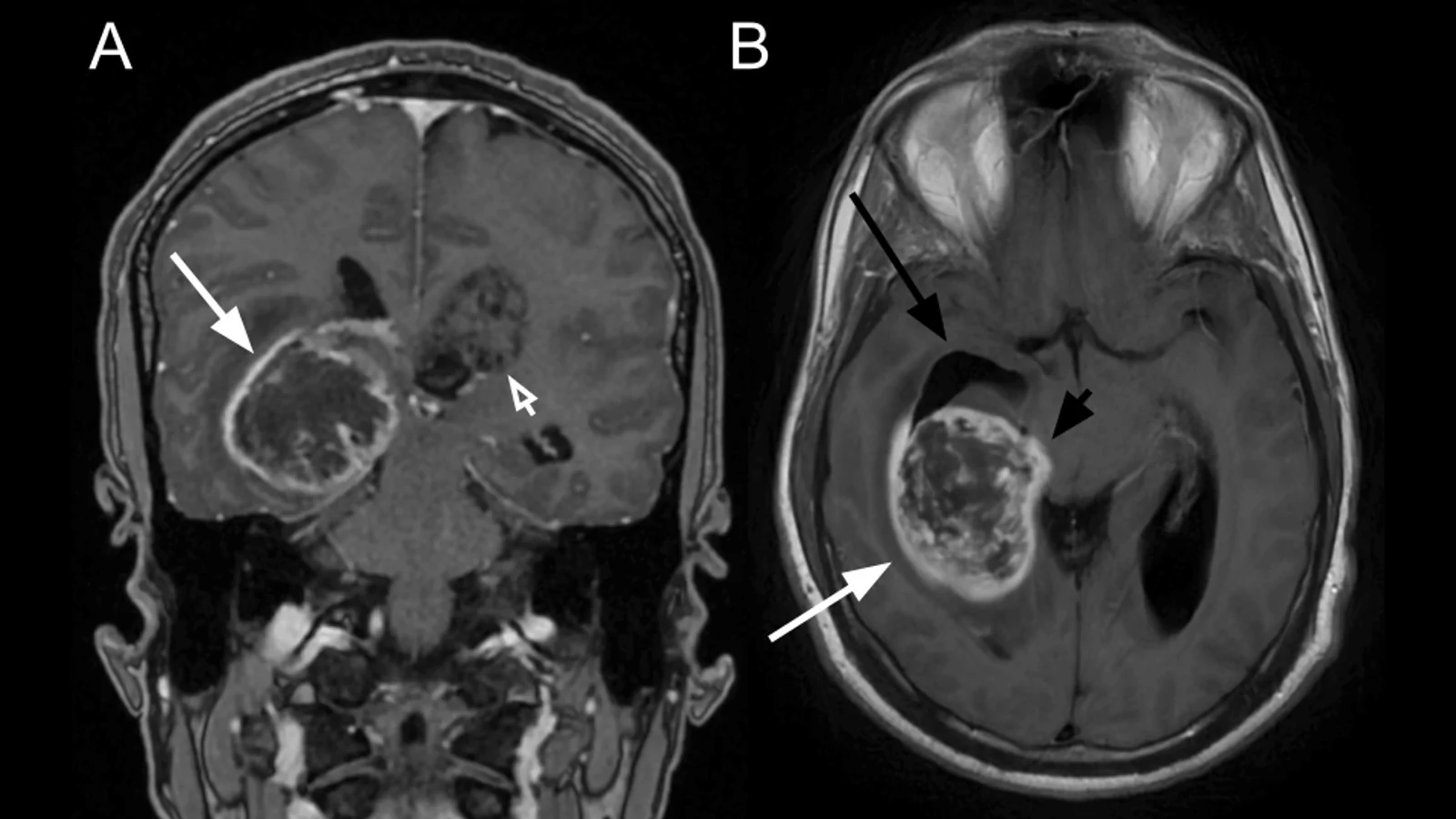
Coronal (Panel A) and axial (Panel B) contrast-enhanced T1-weighted MRI of the brain. Panels A and B demonstrate a heterogeneous T1-weighted peripherally enhancing mass with irregular central lace-like enhancement in the temporal lobe (closed white arrow), consistent with a glioblastoma. In Panel B, associated locoregional mass effect results in asymmetric dilatation of the right temporal horn (long black arrow), concerning for ventricular entrapment with effacement of the basal cisterns (short black arrow). The central neurocytoma in Panel A demonstrates subtle heterogeneous contrast enhancement (open short arrow).

**Figure 3:**
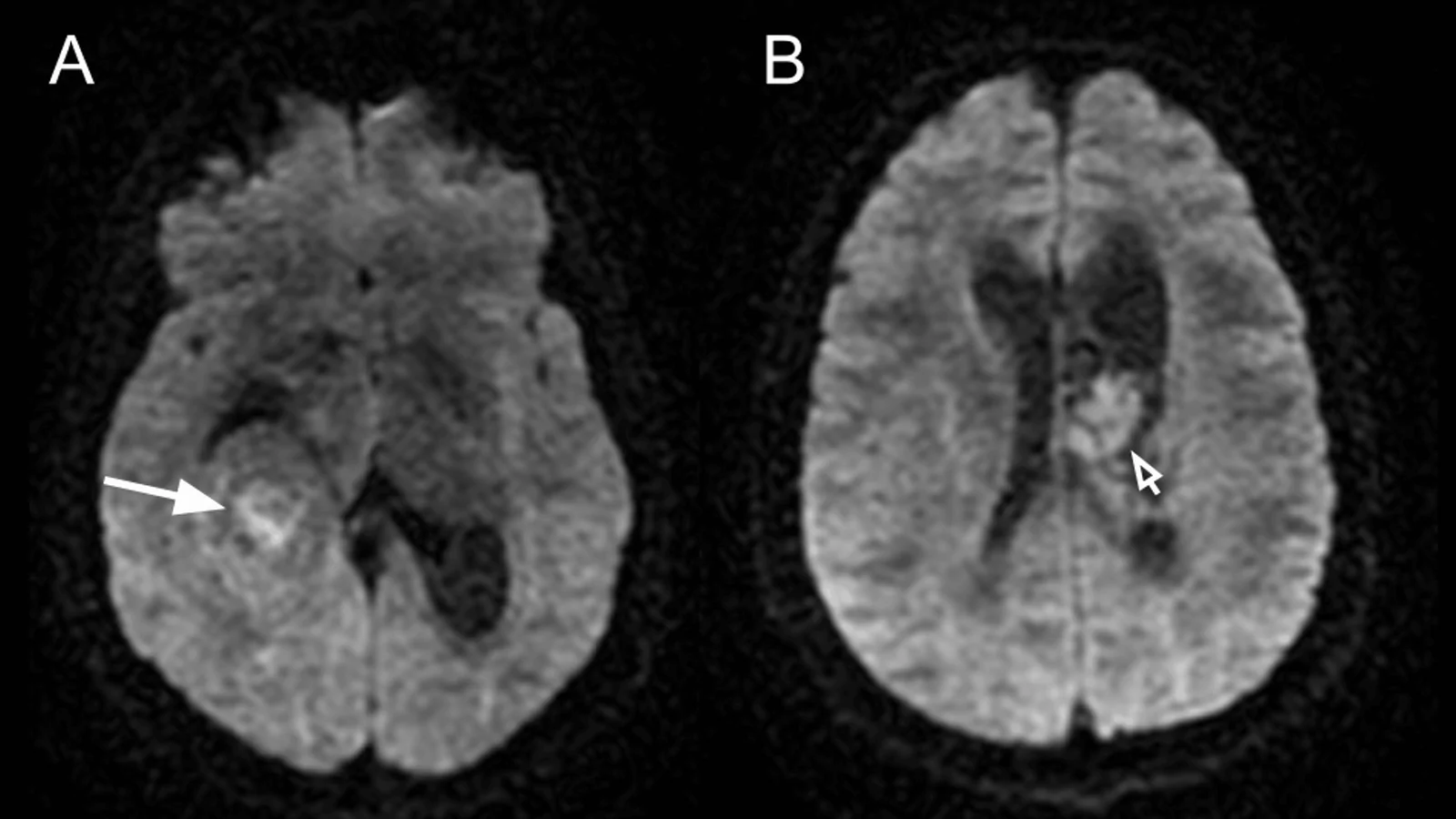
Axial diffusion-weighted MRI of the brain at the level of the glioblastoma (A) and central neurocytoma (B). The closed white arrow in Panel A demonstrates mild central hyperintense signal on diffusion-weighted imaging. Apparent diffusion coefficient (ADC)-weighted imaging (not shown) demonstrated corresponding hypointense signal, consistent with diffusion restriction.  The open white arrow in Panel B demonstrates very subtle heterogeneous hyperintense signal which corresponded to hypointense signal on ADC weighted imaging (not shown), consistent with mild diffusion restriction.

**Figure 4:**
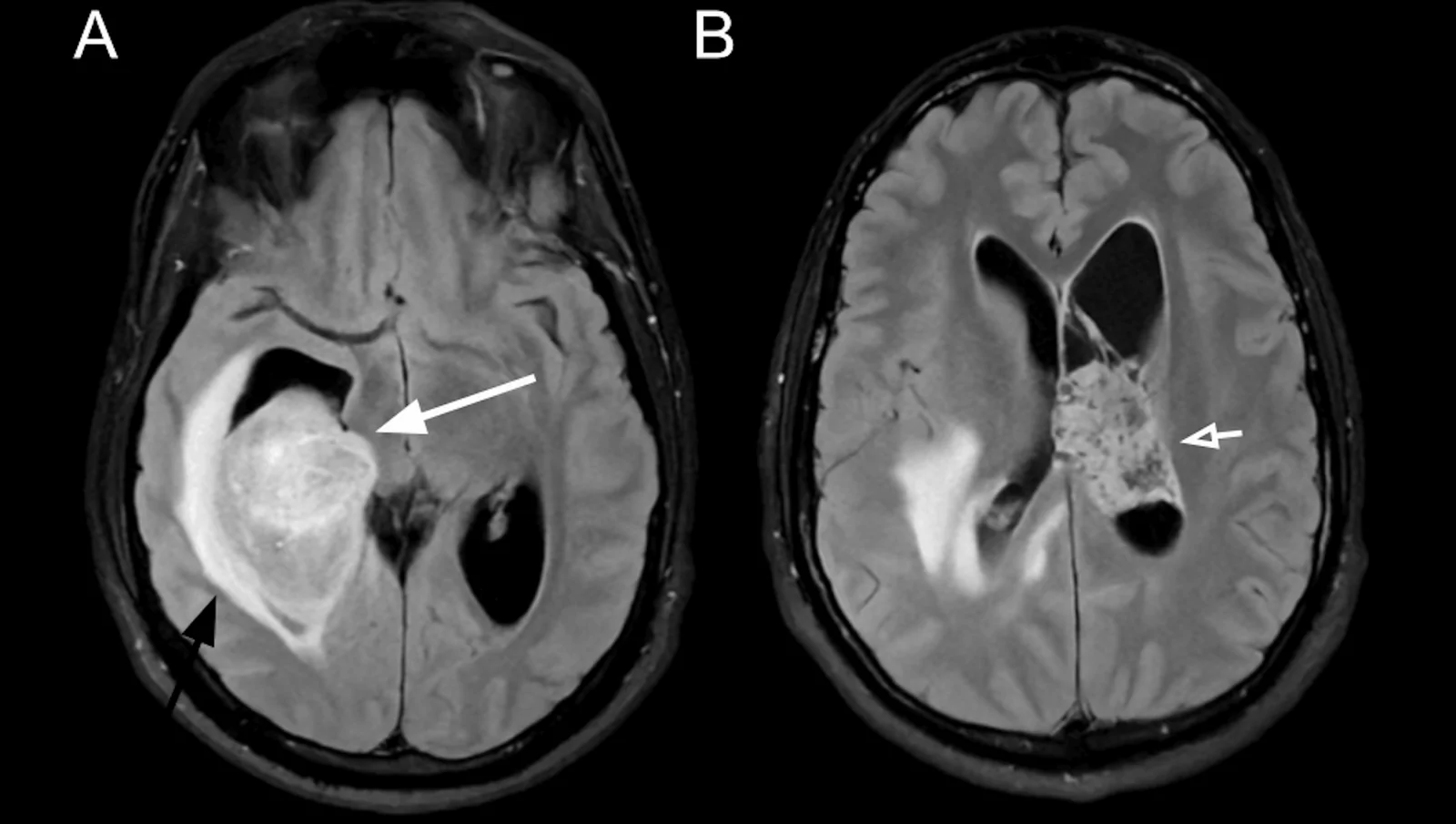
Axial fluid-attenuated inversion recovery (FLAIR)-weighted MRI of the brain at the level of the glioblastoma (A) and central neurocytoma (B). Panel A demonstrates increased T2/FLAIR signal in the glioblastoma (closed white arrow) with peritumoral T2/FLAIR hyperintense signal compatible with vasogenic edema and/or tumoral infiltrate (black closed arrow). Panel B demonstrates a central neurocytoma arising from the septum pellucidum. The lesion is mildly T2 hyperintense with scattered punctate T2 hypointensities corresponding to calcifications (white open arrow), better characterized on CT of the head.

Stereotactic-guided biopsy of both lesions was performed concurrently. Microscopic analysis of the intraventricular lesion demonstrated diffuse sheets of uniform, bland neoplastic cells with round nuclei, granular chromatin, and abundant scattered psammoma bodies. Immunohistochemical analysis was positive for synaptophysin, thyroid transcription factor 1 (TTF-1), and NeuN and negative for chromogranin and Olig2. The Ki-67 index was approximately 2%. The final diagnosis was central neurocytoma, WHO grade 2. Microscopic analysis of the right temporal lesion demonstrated gemistocytic morphology, microvascular proliferation, and extensive necrosis. Immunohistochemical analysis was positive for glial fibrillary acidic protein (GFAP), with retained ATRX expression, and negative for Olig2 and IDH1 R132H. The Ki-67 proliferative index was approximately 25%. The final diagnosis was IDH-wildtype glioblastoma, CNS WHO grade 4.

Following biopsy and multidisciplinary discussion, the patient underwent surgical resection of the glioblastoma. Given the absence of significant neurological symptoms attributable to the approximately 4-cm central neurocytoma, the risks associated with undertaking a second intracranial operation, and the desire to avoid the morbidity of a second procedure, nonoperative management was elected.

Postoperatively, the patient developed mild right-sided visual deficits but otherwise was doing well without other significant neurological deficits. Follow-up imaging obtained two days later demonstrated expected post-resection changes with an adjacent rind of diffusion restriction, favored to represent granulation tissue. The intraventricular neurocytoma remained unchanged in size and appearance. A plan was made for outpatient follow-up with radiation oncology, with a preliminary plan for six weeks of radiation therapy followed by six months of adjuvant chemotherapy.

## Discussion

This case represents a rare synchronous presentation of glioblastoma and central neurocytoma occurring in anatomically distinct regions of the brain. Recognition of characteristic imaging features is important because lesions with similar imaging appearances can have a broad differential diagnosis and may require substantially different management strategies.

In the right temporal lobe, an intra-axial lesion demonstrating heterogeneous enhancement, central nonenhancing mixed-signal-intensity components, diffusion restriction, and extensive adjacent T2/FLAIR hyperintensity can represent several pathologies, including high-grade glioma, glioblastoma, primary CNS lymphoma, particularly in the immunocompromised population, and metastatic disease [3]. In this case, the combination of heterogeneous enhancement with central nonenhancing components, extensive perilesional T2/FLAIR hyperintensity concerning for tumoral infiltration and/or vasogenic edema, and regional mass effect favored an aggressive primary intra-axial neoplasm. In the appropriate clinical and imaging context, these features favored the eventual diagnosis of glioblastoma.

Similarly, a partially calcified intraventricular lesion can have a differential that includes central neurocytoma, subependymal giant cell astrocytoma, and ependymoma [3]. In this case, the lesion was centered in the body of the left lateral ventricle and abutted the septum pellucidum, with a partially calcified appearance, microcystic morphology, subtle heterogeneous enhancement, and subtle diffusion restriction. The characteristic location near the septum pellucidum, together with these imaging features, favored the eventual diagnosis of central neurocytoma.

To our knowledge, only one other case has been reported in the literature. Muragaki et al. described a similar presentation involving an intraventricular central neurocytoma accompanied by two distinct cerebral lesions consistent with glioblastoma [10]. In that study, genotypic analysis suggested a shared clonal origin, and the glioblastomas were therefore interpreted as dedifferentiated metastases arising from the primary intraventricular neurocytoma [10].

In contrast, the findings in the present case favor distinct tumor differentiation. The central neurocytoma demonstrated scattered psammoma bodies and positivity for synaptophysin, TTF-1, and NeuN, supporting neuronal differentiation, whereas the glioblastoma demonstrated gemistocytic astrocytic morphology with positivity for GFAP and negativity for NeuN, supporting glial differentiation. Although these distinct histological and immunophenotypic profiles favor independent tumorigenesis, they do not exclude a shared clonal origin; comparative molecular profiling was not performed and represents an important limitation of this case. Further molecular characterization may help determine whether such synchronous tumors represent independent tumorigenic events or share a common clonal origin.

From a pathogenetic perspective, the coexistence of glioblastoma and central neurocytoma within the same patient remains difficult to explain. Although the coincidental development of two unrelated primary neoplasms is a possibility, alternative mechanisms may account for this rare presentation, including an underlying genetic susceptibility to multiple primary neoplasms or environmental factors contributing to tumor development. Molecular characterization of similar cases may help identify whether these tumors arise through shared biological pathways or represent independent tumorigenic events.

## Conclusions

The synchronous occurrence of two primary intracranial neoplasms is exceedingly rare and highlights the limited understanding of the mechanisms underlying synchronous brain tumors. Additional investigation into the molecular and pathogenetic mechanisms underlying such cases may provide valuable insight into the factors that drive the development of biologically distinct tumors within the same patient.
